# Cognitive impairment in chronic inflammatory demyelinating polyneuropathy

**DOI:** 10.1007/s00415-025-13517-y

**Published:** 2025-11-18

**Authors:** Oliver L. Steiner, Claudia Angela, Elena Krasivskaya, Fabian Klostermann

**Affiliations:** 1 Motor and Cognition Group, Department of Neurology, Charité – Universitätsmedizin Berlin, Freie Universität Berlin and Humboldt-Universität zu Berlin, Campus Benjamin Franklin (CBF), Hindenburgdamm 30, 12203 Berlin, Germany; 2 Berlin School of Mind and Brain, Humboldt-Universität zu Berlin, Berlin, Germany; 3 Institute of Psychology, Humboldt-Universität zu Berlin, Berlin, Germany

**Keywords:** CIDP, Non-sensorimotor symptoms, Cognitive phenotype

## Abstract

**Introduction:**

Cognitive dysfunction has been repeatedly described as an aspect of chronic inflammatory demyelinating polyneuropathy (CIDP), but to which capacities this exactly refers and how it is embedded in the clinical phenotype of the condition remains unknown. Therefore, the detailed cognitive profiles of persons with and without CIDP were compared with each other and the disease-related results analyzed in view of main symptom complexes.

**Methods:**

44 persons with CIDP were studied with respect to their cognitive performances in an extensive computer-based test battery and described clinically in terms of sensorimotor disability (R-ODS), fatigue (FSMC), and mood (DESC-I). The cognitive test results were compared to those of 33 age-matched healthy controls.

**Results:**

The overall cognitive performance of persons with CIDP was significantly worse than that of controls (composite score for CIDP: 33.34 ± 17.96; for Controls: 48.73 ± 18.31; *U* = 382, *p* < 0.001). Pronounced deficits prevailed in processing speed (*q* = 0.018), executive function (*q* = 0.018), memory (short-term: *q* = 0.020; long-term: *q* = 0.025) and visuoconstruction (*q* = 0.018). Using a hierarchical regression model, the inclusion of the factor sensorimotor disability improved the prediction of overall cognitive performance in persons with CIDP (*p* = 0.027).

**Conclusion:**

Cognitive impairment is an under-recognized aspect of CIDP, characterized by reduced processing speed, executive functioning, and memory performance. These cognitive deficits grow together with sensorimotor impairment. The findings show that CIDP goes along with functional changes beyond its classical lead symptoms.

**Supplementary Information:**

The online version contains supplementary material available at 10.1007/s00415-025-13517-y.

## Introduction

Chronic inflammatory demyelinating polyneuropathy (CIDP) is a dysimmune disease targeting the peripheral nervous system (PNS). It is typically characterized by both proximal and distal sensorimotor deficits with progressive or relapsing course [1]. To mitigate paretic symptoms and numbness, its treatment mainly targets the immunological attack against Schwann cells building the axonal myelin sheath around axons [2]. However, many CIDP patients do not only report sensorimotor deficits, but also problems less easily explained as sequelae of the PNS disorder, e.g., fatigue [3, 4] or sleep disturbances [5, 6]. Importantly, these non-sensorimotor symptoms (NSMS) altogether appear to impact on quality of life as much as the lead physical symptoms [4].

Decreased cognitive performance has recently been noticed as a further aspect of NSMS in CIDP [7–9]. Having said that, it remains unclear whether this implies a specific profile of cognitive dysfunction, as known for other neurological conditions (e.g., PD [10]), because the applied tests so far, mainly focused on particular performances in relatively small cohorts [7–9]. Further, it remains to be settled if cognitive task performances are influenced by disease factors, such as physical disability, depression, or fatigue, or if they prevail independently from these aspects in CIDP [11].

In this study, we determined a detailed profile of cognitive functioning in a comparably large group of patients with CIDP, using a computer-based test battery to reduce reliance on motor speed and dexterity for determining exact psychometric data. Furthermore, we assessed clinical and demographic parameters and sought for potential associations with the test data. In so doing, we pursued two questions. First, we aimed at clarifying whether a cognitive profile typical of CIDP could be defined. Second, we sought to gain clues whether cognitive impairment in CIDP, the origin of which remains unknown, was a distinct comorbidity in this condition or if it rather reflected an epiphenomenon of other disease sequelae.

## Methods

### Participants

Forty-four patients diagnosed with CIDP according to the European Federation of Neurological Societies/Peripheral Nerve Society (EFNS/PNS) criteria were initially screened. All of them had a stable disease with unchanged maintenance therapy with intravenous immunoglobulin (IVIg) during the last 3 months. To keep the clinical study cohort uniform in terms of the treatment, patients with other CIDP therapies (e.g., corticosteroids or efgartigimod) were not included. The CIDP type could be typical (symmetric proximal and distal sensorimotor deficits) or a variant (focal, distal-predominant, purely sensory, and purely motor). Additionally, 34 healthy socio-demographically matched controls were recruited. Exclusion criteria for all participants included psychiatric diseases and neurological conditions apart from CIDP. All participants gave written informed consent to the study protocol approved by ethics committee of the Charité–Universitätsmedizin Berlin (EA/165/16) and in line with the Declaration of Helsinki.

### Clinical and psychometric assessments

Cognitive functioning, fatigue, mood, and functional disability were assessed as described below.

### Cognition

For testing the performances in different domains, a computer-based test battery was configured in the Wiener Test system (WTS, SCHUHFRIED GmbH), a standard system for digital psychometric measurements in clinical settings. The assessment covered a wide range of cognitive tasks to provide a detailed profile (see Table 1). Working memory was tested for visual (using components related to the Figural Memory Test, FGT) as well as auditory/verbal modalities (via a Verbal N-Back task). Memory performance was assessed through short-term and long-term recall tasks (related to the FGT). Core executive functions were examined using the WTS modules for the Trail Making Test (TMT A/B) with respect to set-shifting and sequencing, and for the Tower of London (TOL) task in view of planning abilities. Social cognition was probed using the Theory of Mind (TOM) module. Processing speed was measured based on performance in the TMT Part A. Visuoconstructive abilities were assessed with the VISCO test module. Finally, various facets of attention were evaluated, including divided attention (using the WAF cross-modal task) and intrinsic alertness (using the WAF intrinsic alertness task). Performance on the WAF intrinsic alertness task, measured before and after the main cognitive battery, also served as an objective indicator of cognitive fatigability.

**Table 1:** Neuropsychological assessment

Domain	Sub-domain/specification	Test
Working memory	Visual	FGT
	Auditive	-back (verbal)
Memory	Immediate recall	FGT-recall
	Short-term	FGT-recall (short)
	Long-term	FGT-recall (long)
Executive functioning		TMT A/B, TOL
Social cognition		TOM
Processing speed		TMT A
Visuoconstruction		VISCO
Divided attention		WAF (cross-modal)
Subjective cognition complaints		FLei
Depression		DESC-I
Fatigue	Motoric	FSMC motoric score
	Cognitive	FSMC cognitive score, WAF (intrinsic alertness)

DESC-I = Rasch-based Depression Screening version 1; FGT = Figuraler Gedächtnistest; FLei = Questionnaire for Complaints of Cognitive Disturbances; FSMC = Fatigue Scale for Motor and Cognitive Functions; TMT A = Trail Making Test A; TMT B = Trail Making Test B; TOL = Tower of London Test; TOM = Theory of Mind; VISCO = Visuoconstruction Test; WAF (cross-modal) = Wiener Aufmerksamkeitstest (cross-modal)

### Assessment of fatigue, mood, and subjective cognition

Subjective experiences were captured using validated questionnaires. Fatigue severity was quantified using the Fatigue Scale for Motor and Cognitive Functions (FSMC [12]), which assesses both mental and physical fatigue dimensions. Higher scores indicate greater fatigue, with established cutoffs for mild, moderate, and severe levels. Depressive symptoms over the preceding 2 weeks were measured using the Rasch-based Depression Screening (DESC-I [13]), with scores ≥ 12 considered as clinically relevant. Subjective cognitive complaints were documented using the Questionnaire for Complaints of Cognitive Disturbances (FLei). To assess the subjective estimation of sleep quality, we used the Pittsburgh Sleep Quality Index (PSQI), higher values indicating lower sleep quality [14].

### Sensorimotor disability assessment

In the CIDP patient group, functional disability was assessed using the Rasch-built Overall Disability Scale (R-ODS) [14]. This 24-item patient-reported measure evaluates difficulties in daily activities requiring sensorimotor skills (total score 0–48), higher scores reflecting lower disability. Control participants were confirmed to have no limitations in daily living activities.

### Statistics

Statistical analyses were conducted using Python (version 3.12.4) within a Jupyter Notebook (version 7.0.8) environment, employing SciPy [15], statsmodels [16], and Pingouin libraries [17]. The normality of data distribution for each variable within each group was evaluated using the Shapiro–Wilk test. Group differences were analyzed conditionally based on normality outcomes. If both groups showed normal distribution (*p* > 0.05, Shapiro–Wilk test), we applied two-sided independent samples t-tests. Otherwise, we utilized the non-parametric two-sided Mann–Whitney *U* test. Given multiple comparisons across cognitive domains, we controlled the false discovery rate (FDR) using the Benjamini–Hochberg procedure for cognitive subdomains. Statistical significance was defined at an FDR-adjusted threshold of 5% (*Q* = 0.05). Additionally, we computed Bayes factors (BF_10_) corresponding to each performed statistical test to quantify evidence strength. We utilized bootstrapping to calculate robust 95% confidence intervals for differences between group means. Pearson’s and Spearman’s correlations were used to identify factors related to overall cognitive performance. Significant predictors were then entered into hierarchical multiple regression models, with age, sex, and education entered first, followed by functional disability (RODS for patients) and fatigue (FSMC).

## Results

**Fig. 1:**
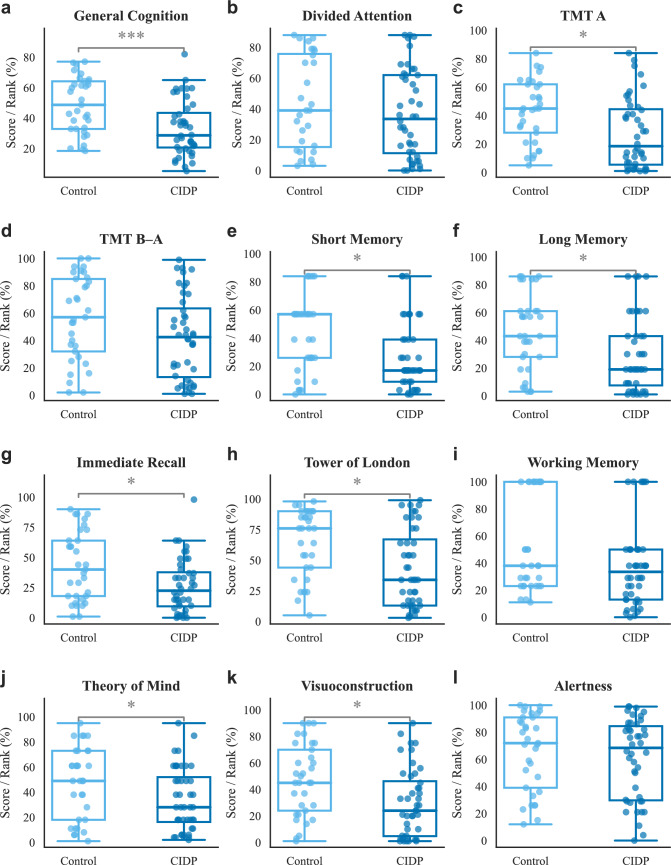
Neuropsychological performance in healthy controls versus CIDP patients across cognitive domains. Age- and education-adjusted percentile ranks (0–100%) are plotted for healthy control participants (light blue) and individuals with chronic inflammatory demyelinating polyneuropathy (CIDP; dark blue). In each box-and-whisker plot, the box spans the interquartile range, the central line marks the median, whiskers show the full data range, and each dot represents one subject. Between-group comparisons were performed with the Mann–Whitney *U* test (*p < 0.05; ***p < 0.001). The comparisons between cognitive sub scores are adjusted for multiple testing with the Benjamini–Hochberg method

**Fig. 2:**
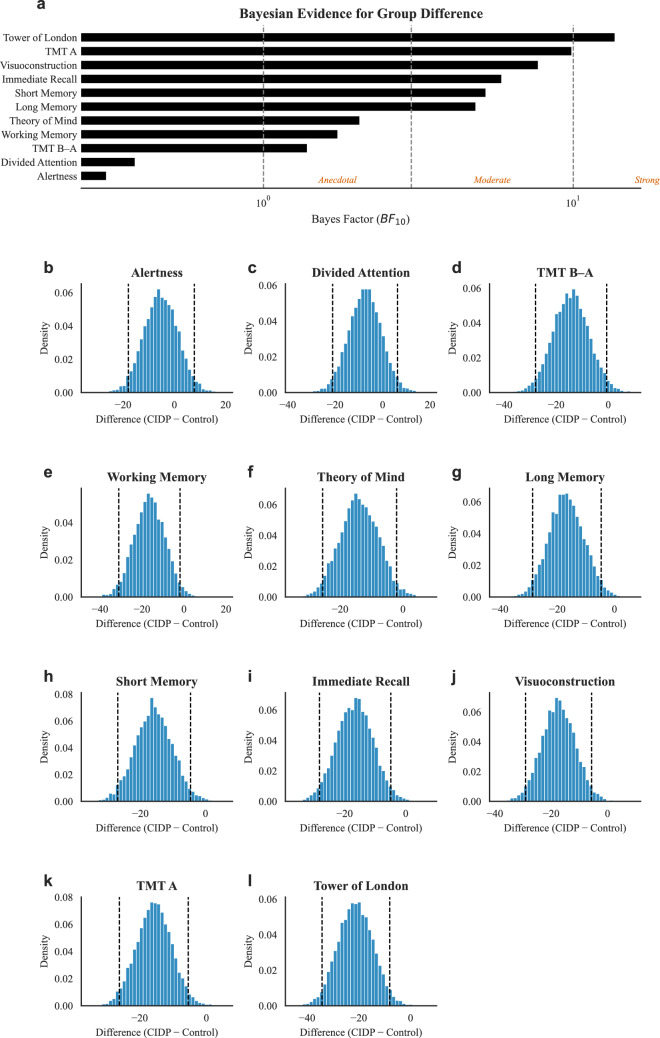
Bayesian comparison of neuropsychological performance in CIDP versus control participants. **a** Bayes factors (BF_10_) for group differences on each cognitive test, shown on a logarithmic scale. Horizontal bars represent BF_10_ values for Tower of London, TMT A, Visuoconstruction, Immediate Recall, Short Memory, Long Memory, Theory of Mind, Working Memory, TMT B–A, Divided Attention, and Alertness (from strongest to weakest evidence). Vertical dashed lines at BF_10_ = 1, 3, and 10 denote thresholds for anecdotal, moderate, and strong evidence in favor of a CIDP–control difference. **b–l** Posterior distributions of the difference in percentile rank (CIDP – Control) for each test. Histograms show the density of sampled posterior estimates; vertical dashed lines mark the 95% credible interval. Negative values indicate lower scores in the CIDP group

Patients and controls did not differ in age, sex or years of education (see also Table 2). Mann–Whitney *U* tests showed lower general cognition in patients (*U* = 382, *p* < 0.001 *q* < 0.001), slower processing speed (TMT A, *q* = 0.018), poorer planning ability (Tower of London, *q* = 0.018), reduced short-term (FGT-short, *q* = 0.020) and long-term memory (FGT-long, *q* = 0.025), diminished visuospatial processing (VISCO, *q* = 0.018) and lower theory‐of‐mind scores (*q* = 0.045; see also Table 3). Bayesian analyses provided convergent evidence (BF_10_ > 5 for all significant contrasts). By contrast, there were no reliable differences in divided attention (WAF), set-shifting efficiency (TMT A/B), working memory (NBV) or alertness (WAF intrinsic); (all *q* > 0.05; BF_10_ < 2, see also Figs. 1, 2. A graphical overview of the cognitive fingerprint of CIDP can be found in the Fig. 3 in the supplementary material).

**Table 2:** Comparison of demographic and clinical characteristics

**Variable**	Controls ( = 33)	CIDP ( = 44)	*p* value
Age (years)	60.6 ± 7.7	63.7 ± 12.8	0.189
Sex (female)	19 (57.6%)	15 (34.1%)	0.068
Education (years)	4.0 ± 1.1	3.7 ± 1.0	0.113
Disease duration (months)	N/A	72.0 ± 65.8	N/A
DESC Score	3.5 ± 3.1	7.6 ± 8.1	0.066
PSQI Score	5.3 ± 2.3	9.0 ± 4.2	< 0.001
FSMC Total Score	34.4 ± 14.3	54.7 ± 23.2	< 0.001
RODS Score	47.0 ± 1.6	35.1 ± 10.5	< 0.001
IVIG dose (g)	N/A	83.3 ± 15.7	N/A
IVIG interval (d)	N/A	33.3 ± 9.4	N/A
CSF cell count	N/A	3.2 ± 3.4	N/A
CSF protein (mg/dl)	N/A	665.1 ± 385.4	N/A

Values are presented as mean ± SD or (%)

CIDP, chronic inflammatory demyelinating polyneuropathy; CSF, cerebrospinal fluid; DESC, Rasch-based depression screening; FSMC, fatigue scale for motor and cognitive functions; IVIG, intravenous immunoglobulin; N/A, not applicable; PSQI, Pittsburgh Sleep Quality Index; RODS, Rasch-built Overall Disability Scale

**Table 3:** Group comparisons between CIDP patients and healthy controls

Cognitive test	CIDP ( = 44)	Controls ( = 33)	*U*	*p*	*q*	BF_10_
	Mean ± SD	Mean ± SD				
Global cognition composite	33.34 ± 17.96	48.73 ± 18.31	382.0	< 0.001	< 0.001	> 100
WAF (divided attention)	36.84 ± 28.57	44.11 ± 30.11	518.5	0.262	0.289	0.39
Trail making test A	27.34 ± 24.43	43.06 ± 21.62	434.5	0.003	0.018	9.90
Trail making test B	42.39 ± 30.37	56.73 ± 30.81	532.5	0.047	0.065	1.39
Figural memory (short-term)	27.30 ± 23.79	43.06 ± 26.32	469.0	0.007	0.020	5.23
Figural memory (long-term)	29.32 ± 26.68	46.15 ± 27.80	487.5	0.014	0.025	4.85
Tower of London	43.02 ± 31.55	64.18 ± 27.83	450.5	0.005	0.018	13.66
Working memory span (-back)	37.23 ± 29.66	53.52 ± 34.82	545.5	0.061	0.074	1.74
Theory of mind assessment	34.05 ± 24.92	47.94 ± 28.13	514.5	0.029	0.045	2.05
Visuoconstruction test	29.61 ± 25.80	47.15 ± 26.91	452.5	0.005	0.018	7.72
WAF (intrinsic alertness)	60.59 ± 29.52	65.85 ± 28.35	640.5	0.381	0.381	0.31

Data are mean ± SD. *p*-values from two-tailed Mann–Whitney *U* tests; *q*-values are Benjamini–Hochberg-adjusted; BF_10_, Bayes factor in favor of H_1_ over H_0_

GenCog, global cognition composite; WAF, Wiener Aufmerksamkeitskeittest; TMT, Trail Making Test; FGT, Figural Gedächtnistest; TOL, Tower of London; NBV, working memory span; TOM, Theory of Mind; VISCO, visuoconstruction; FSMC, Fatigue Scale for Motor and Cognitive Functions

Clinically, patients reported greater overall fatigue and poorer sleep quality (PSQI; all < 0.001). Depression severity (DESC-I) did not differ between groups (*p* > 0.05), see supplementary material Fig. 1).

In patients with CIDP, higher age (*r* = –0.57, *p* < 0.001) and greater fatigue (FSMC; *r* = –0.38, *p* = 0.012) were associated with poorer cognitive performance, whereas higher education (r = 0.52, *p* < 0.001) and better functional status (RODS; *r* = 0.51, *p* = 0.001) correlated with better cognitive outcomes. In healthy controls, cognitive performance was significantly related only to age (*r* = –0.45, *p* = 0.017).

Hierarchical regression in CIDP revealed that age, sex, and education together explained 49% of the variance in a general cognition (model 1). Adding functional disability (RODS) significantly improved the model fit (Δ*R*^2^ = 0.07, *p* = 0.027), while fatigue (FSMC) did not account for additional variance (Δ*R*^2^ = 0.01, *p* = 0.32). In controls, model 1 explained 32% of variance. Adding fatigue (FSMC) and depression (DESC) did not lead to a significantly better explanation of variance (both *p* > 0.13).

## Discussion

In this study, patients and controls engaged in a broad battery of tests to explore the cognitive profile associated with CIDP. Averaged over all tasks, patients performed abnormally low. Next to this overall cognitive deficit, disease-related abnormalities were found in various domains, implying reduced processing speed, executive functioning, memory, visuoconstruction, and theory of mind.

While the identified deficit pattern was considerably broad, certain task performances did not differ significantly between patients and controls. In particular, no abnormalities were seen with respect to working memory, divided attention, and alertness. Of course, it cannot be said that this pattern was specific to CIDP. At the same time, the cognitive deficits did not appear completely unspecific, e.g., as a psychological response to the experience of disease, but they affected some functions preferentially. For example, the TMT results, normal in part B and low in part A, seem indicative of preserved cognitive flexibility, but reduced processing speed, compatible with the idea that the pathophysiology of CIDP targets some brain functions over others. The origin of this selectivity remains speculative. However, a possible mechanism is that the disease is less restricted to the PNS than commonly assumed but regularly implies some subtle neuroinflammation extending to the CNS. In this regard, permeation of inflammatory agents via a leaky blood–brain barrier (BBB) was discussed as a mechanism of pathogenic PNS-to-CNS spread, because CIDP patients with high cerebrospinal fluid protein levels as an indication of a BBB disorder tended to have a higher number of abnormal cognitive test results [8]. Regardless of the mechanisms, recent findings have in fact shown neuronal impairment beyond the PNS in persons with CIDP, for example, as a loss of volume in the ganglion cell and inner plexiform layers of the retina or as lowered functioning of the retinal melanopsin system [5, 18]. Besides, higher levels of serum neurofilament light chain (sNfL), a marker of axonal damage in different neurological conditions, were associated with lower cognitive performance in patients with CIDP, while a link between sNfL and the sensorimotor impairment was no found [19], so that sNfL in CIDP may, at least partially, reflect central axonal impairment. In this regard, it is worthwhile to note that—next to associations with age and education as in other neurological diseases [20]—a negative relation of overall cognitive performance with the degree of sensorimotor disability was identified. This would tie in with the idea of pathophysiological disease progression impacting on PNS and—more subtly—CNS functions in parallel. However, apart from a direct impact on the brain, different theoretical concepts would posit that CNS deafferentiation from peripheral signaling could in itself suffice to induce cognitive change. Depending on the particular framework, embodied cognition models would, for example, imply that the permanent mismatch between input and predictions from sensory representations results in the recruitment of additional computations for the alignment of fuzzy peripheral information with available internal representations [7]. This could, in turn, imply maladaptive effects, e.g., in that the binding and diversion of network resources degraded processing speed and impacted on seemingly unrelated cognitive capacities.

The findings extend and specify previous reports of subtle CIDP-related cognitive abnormalities in circumscribed tasks [7–9]. However, a number of study limitations should be taken into account. First, the used cross-sectional design precludes conclusions about the course of cognitive deficits in CIDP, and longitudinal studies would be needed to determine their evolution. Second, the patients studied in the current trial were under stable IVIg treatment, so that potential effects of available immunomodulatory therapies on cognitive NSMS would have to be addressed in further trials. Third, the presence of cognitive dysfunction in CIDP was assured by using digital psychometric procedures less prone to distortions by sensorimotor, i.e., non-cognitive problems (than paper–pencil testing used in previous studies), but the biological basis of the findings was not addressed. In this regard, study designs with suited techniques, e.g., functional imaging or the assessment of serological biomarkers, could help to disentangle underpinnings of cognitive dysfunction in CIDP. Moreover, while our results point to a pattern of preferential cognitive alterations associated with CIDP, they do not allow conclusions about disease-specificity. In this regard, comparisons with groups suffering from other forms of polyneuropathy would be required. Finally, while the calculated effect sizes for CIDP-related cognitive abnormalities were considerable, their real-world impact, e.g., on social or professional functioning, remains unknown. Better insight into this issue would be desirable for optimal patient counselling, and future research in this field could provide clinically relevant information.

In conclusion, this study provides robust evidence for a broad range of cognitive abnormalities in persons with CIDP, growing with the degree of sensorimotor disability. Increased awareness of this could result in a more comprehensive treatment of patients with disease sequelae beyond the lead signs of the disease.

## Supplementary Information

Below is the link to the electronic supplementary material.

Supplementary file1 (PDF 66 kb)

Supplementary file2 (PDF 71 kb)

Supplementary file3 (PDF 50 kb)

## Data Availability

The anonymized source data of this study are available upon request.
