# Supplementary material for: Cognitive impairment in chronic inflammatory demyelinating polyneuropathy
Source: J Neurol. 2025 Nov 18;272(12):769. doi: 10.1007/s00415-025-13517-y (PMC12627132; doi:10.1007/s00415-025-13517-y)
Supplement: Supplementary file 1 — Supplementary file1 (PDF 66 kb) [file 415_2025_13517_MOESM1_ESM.pdf]

Supplementary Figure 1: Clinical Score Comparisons

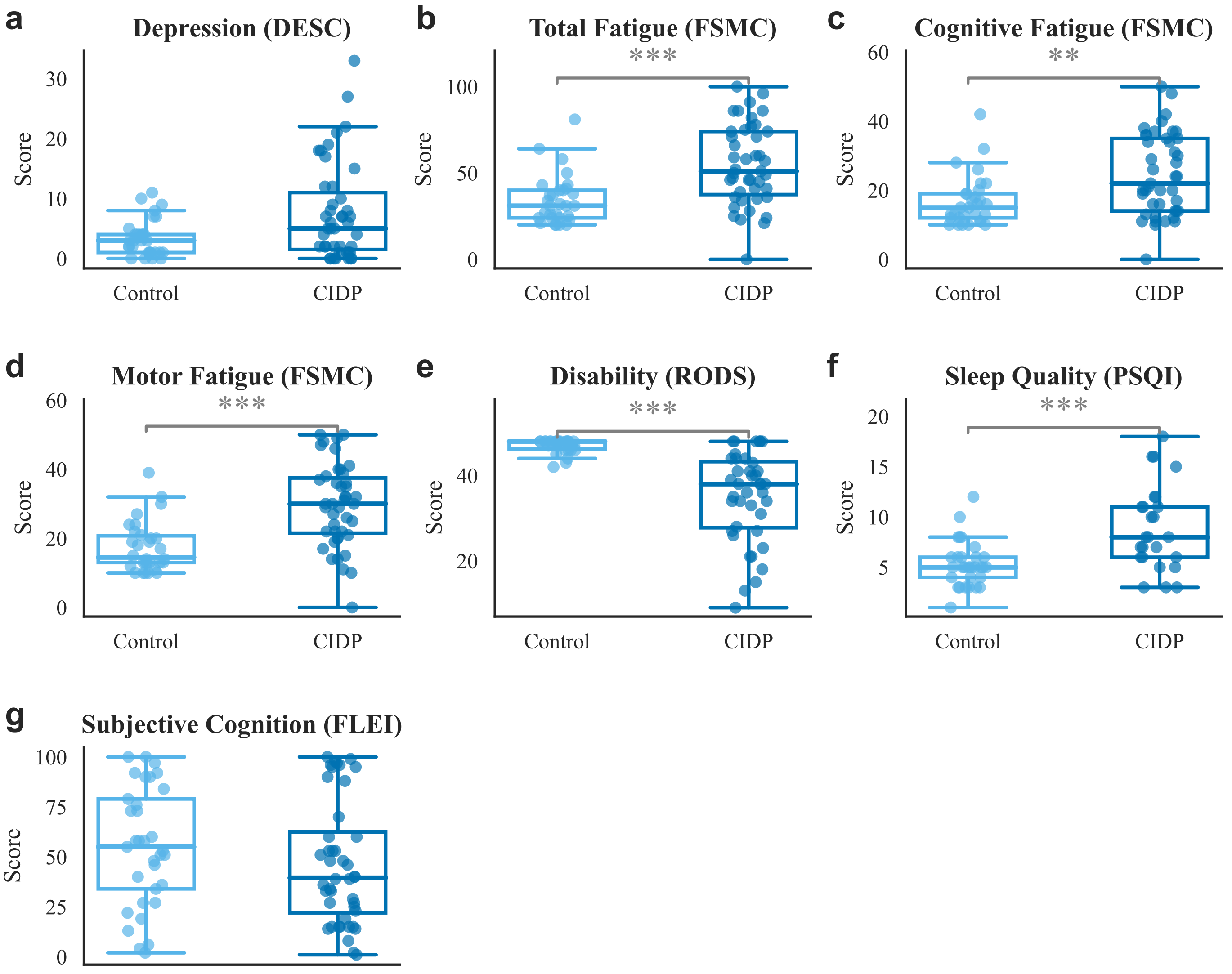

Supplementary Figure 1. Comparison of clinical scores between healthy controls (light blue) and CIDP patients (dark blue). Scores for depression (DESC), fatigue (FSMC), sleep quality (PSQI), and subjective cognition (FLEI) are higher in patients, while quality of life (RODS) is lower. Individual data points are overlaid on boxplots. Asterisks denote significance after FDR correction.
