# Supplementary material for: Cognitive impairment in chronic inflammatory demyelinating polyneuropathy
Source: J Neurol. 2025 Nov 18;272(12):769. doi: 10.1007/s00415-025-13517-y (PMC12627132; doi:10.1007/s00415-025-13517-y)
Supplement: Supplementary file 2 — Supplementary file2 (PDF 71 kb) [file 415_2025_13517_MOESM2_ESM.pdf]

# Supplementary Figure 2: Subjective Cognition vs. Objective Performance in Patients

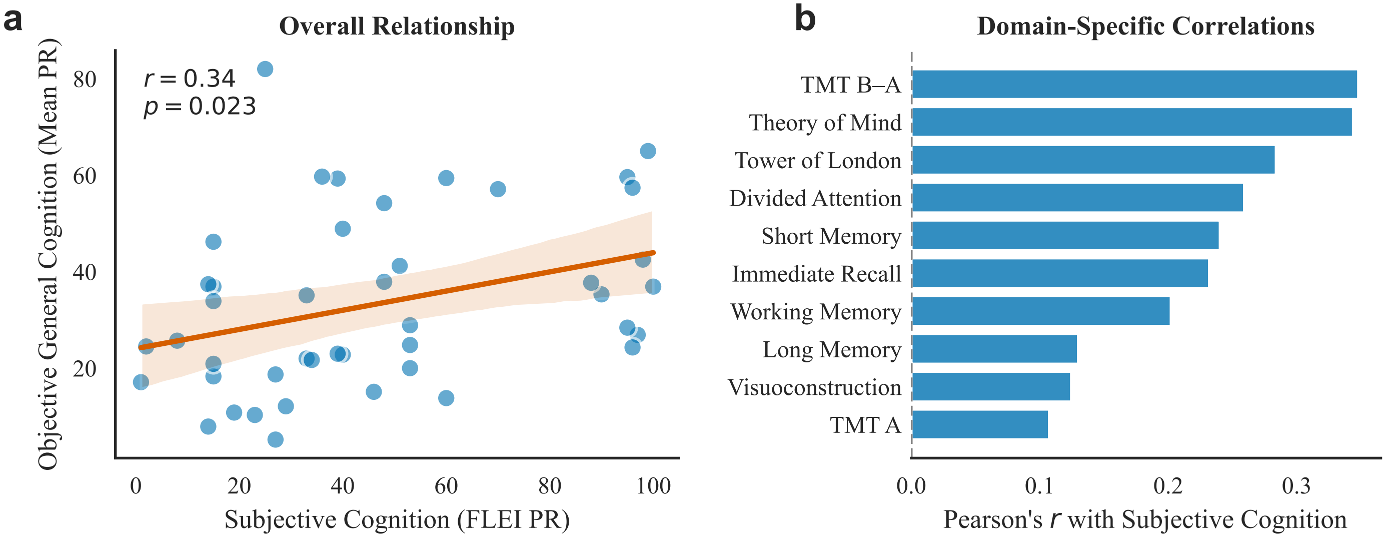


*Supplementary Figure 2. (a) Scatter plot showing the relationship between subjective cognitive complaints (FLEI) and objective general cognitive performance (GenCog) in patients, with the line of best fit. (b) Horizontal bar plot showing the Pearson's r correlation between subjective complaints and each individual objective cognitive domain, sorted by correlation strength.*
