# Supplementary material for: Cognitive impairment in chronic inflammatory demyelinating polyneuropathy
Source: J Neurol. 2025 Nov 18;272(12):769. doi: 10.1007/s00415-025-13517-y (PMC12627132; doi:10.1007/s00415-025-13517-y)
Supplement: Supplementary file 3 — Supplementary file3 (PDF 50 kb) [file 415_2025_13517_MOESM3_ESM.pdf]

Supplementary Figure 3: Cognitive Fingerprint

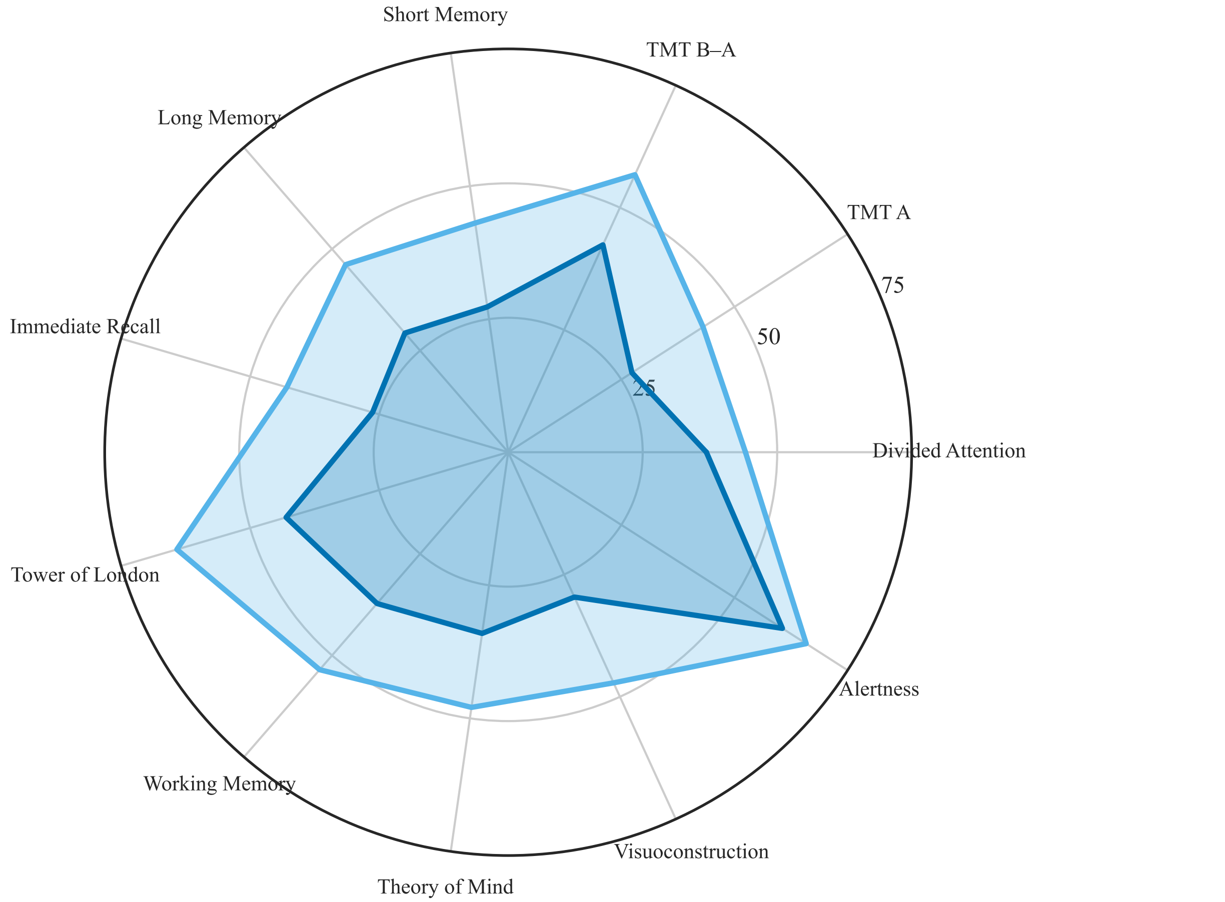

Supplementary Figure 3. Radar plot visualizing the mean cognitive profile ('fingerprint') for CIDP patients (dark blue) relative to controls (light blue) across all tested domains. The radial axis represents percentile rank (PR), showing the domains most affected in the patient group. The legend distinguishes the two groups.
